# Classification of flood-generating processes in Africa

**DOI:** 10.1038/s41598-022-23725-5

**Published:** 2022-11-07

**Authors:** Yves Tramblay, Gabriele Villarini, Mohamed Elmehdi Saidi, Christian Massari, Lina Stein

**Affiliations:** 1 HydroSciences Montpellier (University Montpellier, CNRS, IRD), 300 Avenue du Professeur Emile Janbreau, 34090 Montpellier, France; 2 IIHR-Hydroscience & Engineering, The University of Iowa, Iowa City, IA USA; 3 Georesources, Geoenvironment and Civil Engineering Laboratory, Cadi Ayyad University, Marrakesh, Morocco; 4 Research Institute for Geo-Hydrological Protection, National Research Council, Perugia, Italy; 5 Institute of Environmental Science and Geography, University Potsdam, Potsdam, Germany

**Keywords:** Climate sciences, Hydrology

## Abstract

River flooding has large societal and economic impacts across Africa. Despite the importance of this topic, little is known about the main flood generating mechanisms in Africa. This study is based on 13,815 flood events that occurred between 1981 and 2018 in 529 catchments. These flood events are classified to identify the different flood drivers: excess rains, long rains and short rains. Out of them, excess rains on saturated soils in Western Africa, and long rains for catchments in Northern and Southern Africa, are the two dominant mechanisms, contributing to more than 75% of all flood events. The aridity index is strongly related to the spatial repartition of the different flood generating processes showing the climatic controls on floods. Few significant changes were detected in the relative importance of these drivers over time, but the rather short time series available prevent a robust assessment of flood driver changes in most catchments. The major implication of these results is to underline the importance of soil moisture dynamics, in addition to rainfall, to analyze the evolution of flood hazards in Africa.

## Introduction

African countries are highly vulnerable to floods, with several studies reporting an increase in mortality rate and exposure in recent decades^1–5^**.** A better understanding of the dominant flood-generating mechanisms across Africa is therefore paramount to improve flood forecasting, leading to higher resilience to this natural hazard. The attempts of the hydrologic scientific community to detect changes in flood frequency have warned about existing and well identifiable trends, even though the direction of these trends is not always consistent among the studies^6,7^. A possible explanation for these differences is that the flood records were not examined through the lenses of flood generating mechanisms, which can vary within the same river basin and over time^8,9^. While the lack of representative data sets over the African continent prevented a thorough analysis of floods at the continental scale, a recent database leveraging several data sources now makes such analysis possible^10^.

Several classification methods have been proposed to analyze flood generating mechanisms^11–13^, both on national^14^ and global scales^9^. The flood generating mechanisms, or drivers, are causative classifications of flood events based on hydrometeorological variables (e.g., rainfall, temperature) observed within catchments, the catchment state (e.g., snow depth, soil moisture), and hydrological processes (e.g., infiltration or saturation excess) leading to floods^12^. The most important flood generating mechanisms, or processes, include short heavy rainfall events, long heavy rainfall events, rainfall excess, snow melt and ice jamming^9,11,12,15^. Even though flood-generating mechanisms cannot be defined unequivocally^12^, the 90th, 95th or 99th percentiles of the rainfall distribution are commonly used to characterize extreme rainfall events^16–18^. Conversely, a fixed soil moisture threshold for flood generation is not representative of all the different catchment conditions and different values have been reported in the literature for wet soil conditions^19–24^. Indeed, the nonlinear storage-discharge relationship may be catchment dependent resulting in variable soil moisture threshold percentiles to identify wet and dry conditions^14^.

To apply a flood-event classification at the African scale, a trade-off between classification complexity and robustness should be sought due to the uncertainties in data and a lack of detailed knowledge on flood processes in many of these regions. Uncertainties in precipitation products without a reliable ground-based rainfall reference network over Africa, especially for extreme events^25^, complicates classification. Indeed, it is usually considered that satellite and reanalysis products are better at detecting the rain occurrence than its intensity^26^. Consequently, a robust flood classification for Africa should rather rely on metrics such as the number of consecutive rainfall days or exceedances of predetermined thresholds, rather than on rainfall intensity or estimated quantities such as the runoff coefficient or soil water balance. Not only these quantities are highly sensitive to their estimation method, notably to estimate base flow^22^ or event durations to extract direct runoff^27,28^ but also to the bias in either discharge or rainfall data^29^.

Here we provide a process-based classification of flood events led by three drivers: (1) excess rain, (2) long rain and (3) short rain, across a wide range of river basins in Africa. We use the classification method proposed by Stein et al.^9^, which was previously applied globally but adapted herein to better account for the effects of antecedent soil moisture prior to the flood peaks. In Tramblay et al.^10,30^, it was shown that in most African river basins the annual flood is more strongly associated with soil moisture rather than rainfall extremes. However, as this analysis was based on dominant drivers only, it was not able to detect the relative influence of the different flood drivers. In the present study, we aim to fill this knowledge gap by answering two main questions: (i) what are the contributions of different flood generating processes in Africa? and (ii) are these drivers changing over time?

## Data and methods

### A pan-African river discharge dataset

We use 529 river discharge time series^10^, with at least 10 years of full daily data available during the time period 1981–2018. They cover different basins spanning a wide range of hydro-climatic conditions in Africa, from humid equatorial areas to arid areas (see Supplementary Fig. S1). The highest station density is found in Northern, Western and Southern Africa, while Eastern and Central Africa have a lower density of stations. The median catchment size is 1400 km^2^, with a range from a few square kilometers to 3 × 10^6^ km^2^ for the Congo River basin at Brazzaville, the largest basin in this catalog. Most of the basins are smaller than 20,000 km^2^, with only 118 basins (22% of the total) exceeding this size; therefore, the sample of catchments considered here mostly represents small to moderate basin sizes (78%). Daily rainfall is extracted for the selected catchment from the ERA5 reanalysis^31^, together with daily soil moisture from ERA5-Land^32^. Soil moisture from the ERA5-Land second layer is transformed to a Soil Wetness Index by normalizing daily values by the long-term maximum and minimum of the series. To document the catchment properties, we also extracted land cover, elevation and mean climate characteristics (i.e., mean rainfall, potential evapotranspiration and Aridity Index) from the African Database of Hydrometric Indices^10^. The same methodology was applied with a different rainfall dataset with a higher spatial resolution, the Climate Hazards Group InfraRed Rainfall with Station data (CHIRPS) and the same results were obtained, indicating that the selection of the rainfall dataset has little influence on the results presented here.

To document the effects of river regulation, the number of dams within each basin was extracted from the Grand Dam database^33^. In addition, we used the Degree of Regulation (DOR) computed at the reach level^34^, which is equivalent to the residence time of water in the reservoir and calculated as a ratio between the storage capacity and the total annual flow from the WaterGAP model^35^ between 1971 and 2000. Note that the identification of potential regulations of river basins has large uncertainties in Africa due to incomplete or inconsistent metadata (e.g., the year of dam build, storage capacity, area draining to the reservoir) that could influence the metrics (such as the DOR) used to quantify the effects of dams. For instance, Sadaoui et al.^36^ reported that there are 101 dams in North Africa, while the GrandDam database only includes 53 for the same area. The impact of dams on floods is complex and equivocal. While dams generally reduce flood magnitude^37,38^, some dam management rules cause artificial floods to sustain water use for fisheries and agriculture downstream of several African basins, notably in West Africa^39,40^. To overcome this problem, we did not exclude a priori regulated basins and analyzed the results considering the presence/absence of dams and the DOR. This is consistent with previous studies showing the difficulty of detecting the impact of river regulation on flood changes^3,41,42^.

### Identification of catchment-scale soil moisture thresholds

The approach to identify catchment-specific soil moisture thresholds is based on a sample of runoff events (i.e. not only floods) with matched rainfall and soil moisture data. The runoff events for a given catchment are extracted following these two steps:

1. To avoid the detection of false events caused by small river discharge fluctuations, only daily discharge values higher than the 10th percentile were considered as potential events^28^. Runoff events with no recorded rainfall in the previous days were discarded.
2. De-clustering to identify single events (and not introduce autocorrelation in the analysis), using two rules^16^: a minimum of *n* days between events, with *n* = 5 + log (catchment area) and between two consecutive peaks, discharge must drop below 2/3 of the smallest peak. The maximum discharge of each event is kept.

From this dataset of runoff events of different magnitudes, the event-based rainfall is extracted. Event-based rainfall is estimated by a cumulative sum of rainfall before the date of maximum discharge for each event, and this aggregation stops if a day has zero rainfall. Other thresholds to define zero rainfall (1 mm, 2 mm) have been preliminarily tested without effects on the results. Then, the antecedent soil moisture is extracted for the day before the start of the rainfall event. The Spearman correlation between antecedent soil moisture and event runoff is computed. An exponential model is fitted to the soil moisture/runoff relationship, as previous studies identified this form of dependence^14,19,21,43^. To identify a potential inflexion point in the soil moisture/runoff relation (i.e., the max slope of the curve), we apply the Pruned Exact Linear Time method^44^ to detect a change point (i.e., the soil moisture threshold) in the exponential fit. This procedure is applied only for the cases when there is a significant correlation (at the 5% level) between runoff and soil moisture, to avoid the detection of a change point in cases where there is no relationship between these two variables.

### Classification of flood events

The classification is applied to a sample of flood events corresponding to a mean occurrence of one event per year. This type of sampling is chosen since low or zero annual maxima discharge could be observed in semi-arid and arid areas^45^. In addition, a flood sampling based on extreme rainfall would be likely to overestimate the influence of rainfall, while episodes of moderate rain but on wet soils are also likely to generate floods. The classification is adapted from a previously implemented classification at the global scale (Stein et al.^9^) and over the United States (Stein et al.^46^), in the present study the soil moisture threshold is estimated for each catchment as described in the previous section. The classification relates the occurrence of rainfall amounts above various thresholds to the occurrence of floods. Therefore, even if a flood peak magnitude is reduced due to the presence of a flood-mitigation structure (e.g., dam), its occurrence remains unchanged and the approach is able to detect its driver.

Flood events in each catchment are classified according to three hydrometeorological generating processes, namely, the excess rainfall, short rainfall, long rainfall using a decision tree (see Supplementary Fig. S2)^9,46^. Excess rainfall event is defined as a flood event triggered by rainfall above average (7-day) occurring over saturated soils (above soil moisture thresholds defined in Section “Identification of catchment-scale soil moisture thresholds”), short rainfall as a single day rainfall event above high thresholds (the 90th percentile) and long rainfall as several consecutive days with rainfall above the 90th percentile of rainfall summed over 7 days. The rainfall percentiles have been computed only on wet days. The same classification has been applied using the 95th percentiles as a threshold instead, providing equivalent results. The hydrometeorological conditions are evaluated in a 7-day time period before each flood event, using the date of the flood event, rainfall and soil moisture data. We consider a time period of seven days before the flood events to be consistent with previous studies^9,47,48^ and therefore allows for the comparison across regions. The decision tree first evaluates if a larger-than-average multi-day rainfall fell on previously saturated soil to determine if the flood event was an excess rainfall type of flood. If that was not the case, it evaluates whether the thresholds for long rainfall and then short rainfall are exceeded. If no process could be identified, the class “other” is assigned. Snowmelt events are not considered because there are no basins influenced by snow.

### Changes in floods generating mechanisms

Only for time series longer than 20 years (345 basins), we split the records into two periods to assess the changes in the flood drivers^49^. However, it should be recalled here that the available African discharge time series are generally shorter than those available in Europe or the United States, which limits the robustness of this analysis herein. Since this procedure is based on the comparison of two frequency counts, we use a variant of the Chi-square test^50^ to identify the catchments where the distribution of flood drivers may be significantly different between the two time periods. In addition, we also analyzed the trends in flood-event total rainfall (rainfall summed over 7 days prior to floods), maximum rainfall (maximum daily rainfall recorded during a 7-day period before floods) and antecedent soil moisture condition. While several studies have been focused on trend detection in flood magnitude, some studies also analyzed changes in flood drivers^48,51,52^ but none at the scale of in Africa. Thus, this analysis is useful to understand the potential changes in terms of rainfall intensity or soil moisture that can influence the genesis of floods. For trend detection, we applied the Mann–Kendall^53^ test adapted to potential autocorrelation in the time series and the Sen slope estimator to get a quantitative estimate of the slope of the detected trends (expressed as a relative change to the mean).

## Results and discussion

### Soil moisture thresholds

**Figure 1:**
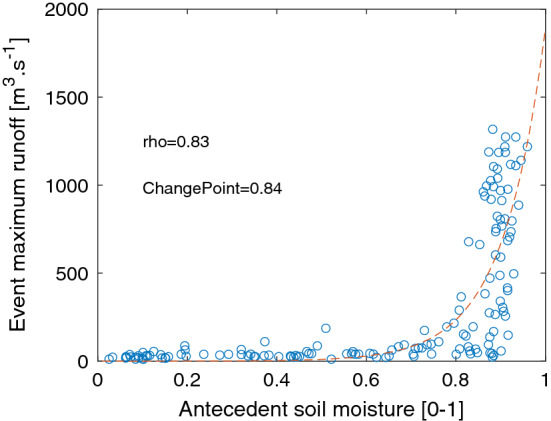
Example of the relationship between antecedent soil moisture and events maximum runoff (Sankarani River in Guinea, 22,102 km^2^). The correlation between event maximum runoff and antecedent soil moisture is equal to 0.83 and the change point identified in antecedent soil moisture is equal to 0.84.

**Figure 2:**
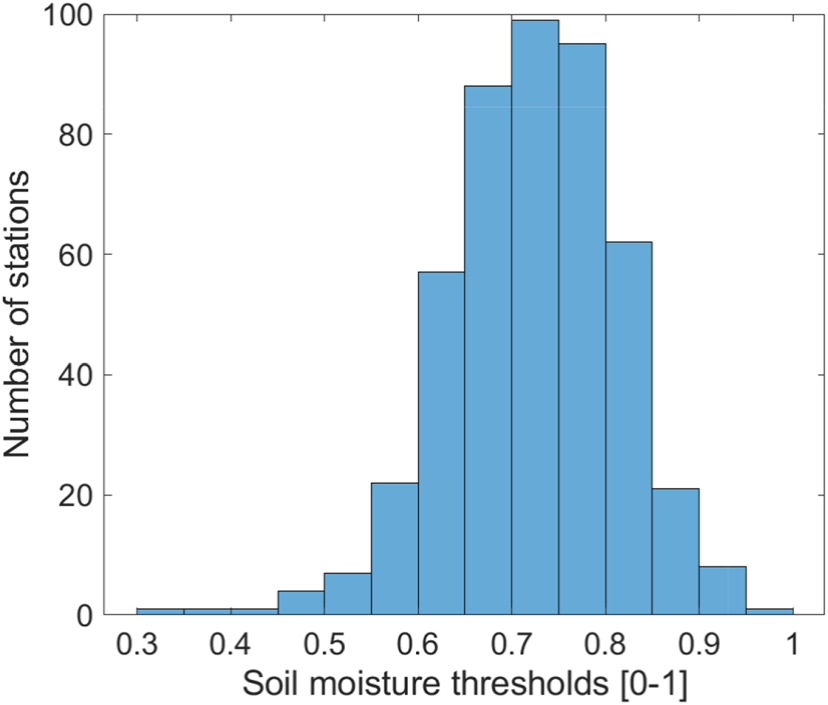
Histogram of soil moisture thresholds identified for all basins.

The first step of the analysis consists in estimating the relevant soil moisture thresholds for runoff generation. On average, the median Spearman correlation between runoff events and antecedent soil moisture is equal to 0.54. An example of the relationship obtained between event maximum runoff and initial soil moisture conditions is shown in Fig. 1; in the majority of the cases there is good fit with an exponential model, consistent with findings from other regions^19,21,28,29^. For most basins, the identified threshold for soil moisture is below 0.9, and close to 0.7 (Fig. 2). The soil moisture threshold is significantly correlated with the Aridity Index (i.e., the ratio of average rainfall to potential evapotranspiration) of the basins (rho = 0.42), with lower thresholds for the semi-arid basins, and no influence of soil moisture on runoff for the most arid ones, consistent with experimental results^54^. The soil moisture thresholds are also higher in larger basins, with a weak but significant correlation with basin size (rho = 0.23). For 82 stations (15%) there is no correlation between soil moisture and runoff events: these stations are among the most arid in the database, with 75% of them having an aridity index below 0.5 (i.e., semi-arid to arid areas) and located mostly in Northern and Southern Africa. For these basins, the soil moisture threshold used in the flood driver’s classification is set to infinity to not classify the floods of these basins in the class “Excess rain.” There is no evident link with river regulation, given that the degree of regulation (DOR) is equal to zero for 67 out of these 82 basins (68%). These findings are fully consistent with those obtained by previous studies in different parts of the globe^19,21,23,24,55,56^ using most often the runoff coefficient rather than maximum discharge during an event to estimate the soil moisture thresholds.

### Spatial distribution of flood generating processes

**Figure 3:**
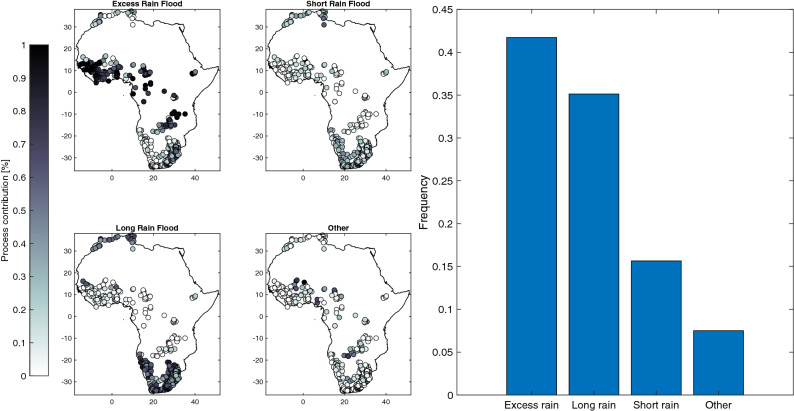
Results of the flood event driver’s classification. The left panels are showing the relative contribution of the different flood-generating process for each basin, the histogram on the right shows the relative importance of each flood generating processes for all basins.

**Figure 4:**
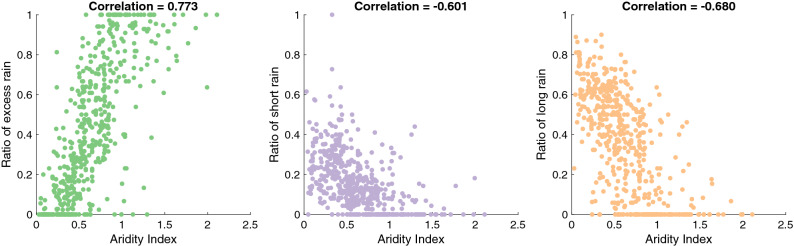
Scatterplots and Spearman correlations between the ratio of excess rain, short rain and long rains with the aridity index for the 529 catchments. All the results are significant at the 1% level. Aridity index is defined as AI = P/PET, so higher (lower) AI values correspond to a more (less) humid climate.

The results of the flood events classification applied to a total of 13,815 flood events are shown in Fig. 3. The two main dominant flood processes are excess rain and long rains, which together represent more than 75% of flood events across all basins. The maximum floods (i.e., the maximum of the flood samples) follow the same distribution, with 231 basins having their maximum flood associated with excess rain and 212 with long rains. The degree of aridity exerts a strong control on the proportion of the different flood classes (Fig. 4), with a greater ratio of excess rain in more humid basins (rho = 0.77) and more frequent short and long rains in the most arid ones (rho = − 0.6 and rho = − 0.68, respectively)^82^. The proportion of long rains gets higher as the climate gets dryer, since soil moisture remains low most of the time, while we see the opposite for excess rains. There are also some significant associations with drainage areas, with a percentage of flood driven by excess rainfall greater in larger basins (rho = 0.21). On the contrary, the correlations are negative between the basin’s areas and the percentage of short (rho = − 0.3) or long rain events (rho = − 0.36), indicating that rainfall-driven floods tend to be more frequent in smaller basins. Overall, these results indicate strong interplays between both the catchment area and the level of aridity to explain the relative influence of rainfall-driven floods. Furthermore, there is no correlation between the frequency of flood events classified as “other” and the degree of regulation of the basin, indicating that the classification of flood events as “other” may be more related to data quality issues.

Two main spatial patterns in terms of flood generating mechanisms can be identified. The first group includes basins in Western and Central Africa where excess rainfall over saturated soil is the main driver. For the most humid basins of this region, the proportion of excess rain events exceeds 90%. The second group includes basins where long rain events are the major flood driver. These stations are mainly located in North and South Africa, under semi-arid to arid climate conditions. In North Africa (North of 25°N), there is a larger proportion of long rain (46%), followed by excess rain (28%), with 26% of short rain, on average for all basins. In Southern Africa, long rains are also the main driver (52%), followed by excess rain (28%) and short rain (20%). We see in the spatial distribution of flood generating processes that the main factor explaining this distribution is the climate, similar to what is found in other parts of the world^42,57^, but modulated at the local scale by the characteristics of the basins.

These results exemplify the importance of soil moisture conditions, either before the beginning of the rainfall event in case of excess rain or during the flood event in case of long rain. Indeed, in many semi-arid or arid environments, the high proportion of sandy soils with high hydraulic conductivities are leading to a fast decay of soil moisture following a rainfall event^58,59^. In these basins, the relevance of antecedent soil saturation for flood generation is seasonal rather than event-based, with higher runoff coefficients during the dry than the wet season due to the reduced development of vegetation and crusted surfaces^60–62^. Consequently, it is not the antecedent soil moisture that plays a role for flood generation in these basins, but a rainfall event lasting several consecutive days which has the potential to gradually saturate the soil during an event^63–65^. The relatively low proportion of short rains in the classification could be partly explained by the daily step of our analyses, which prevents representing flash flood events triggered by short but intense rainfall^66–69^. Therefore, it is likely that the importance of short rainfall events is underestimated in the present work due to this limitation. To overcome this, high-resolution satellite rainfall products could be used^66,70^, even though the results could be affected by their relatively short record length.

### Temporal evolution of flood generating processes

**Figure 5:**
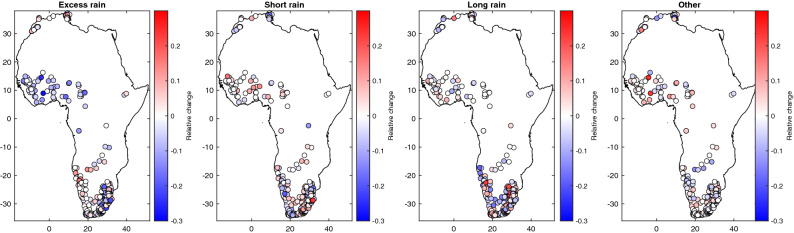
Changes in flood generating processes over time. Red (blue) colors indicate an increase (decrease) in the frequency of occurrence for a given flood trigger in the second period.

**Figure 6:**
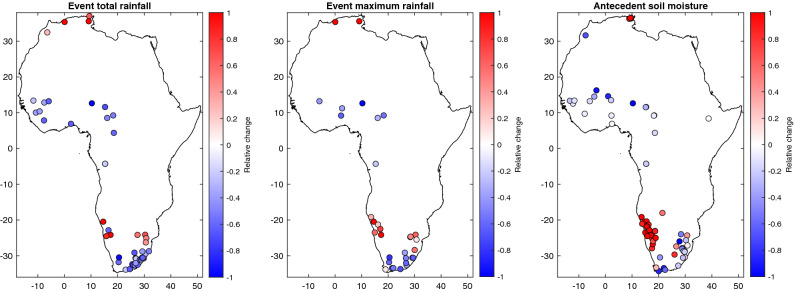
Trends in flood-event total rainfall, event maximum rainfall and antecedent soil moisture. Only trends significant at the 5% level are reported on the map.

The analyses in the previous section provide a climatological view of the major flood drivers. Here we examine whether the dominant flood-generating mechanisms change over time. Overall, little changes in the proportion of each flood generating process in the different basins are detected (Fig. 5), consistent with the results also obtained with similar methods over Europe^49^. On the contrary, increased flood extents in central Europe and the British Isles related to an increased proportion of excess rains over saturated soils have been reported^71^. When examining trends in flood drivers (Fig. 6), significant trends in event total rainfall are detected in 47 stations only (13% of the total), event maximum rainfall in 32 stations (9%) and antecedent soil moisture in 67 stations (19%). The trends detected in these two flood drivers are consistent with the evolution of rainfall, notably with an increase of rainfall extremes in South-Western Africa in the relation with an increased moisture transport from the Namibia low-level jet^3^. For antecedent soil moisture, a large increase is also observed in this region while in Western-Africa several stations exhibit a decrease.

In Africa, for 85% of the basins the changes in the relative proportion of flood drivers are lower than 10% for all categories. For excess rain, in 12 basins there is an increased frequency between + 10 and + 15%, and conversely a decrease in 44 basins between − 10% and − 20%. For short rain, there is an increase in 19 basins between + 10% and + 22%, and a decrease in 23 in basins between − 10% and − 25%. For long rain, there is an increase in 19 basins between + 10% and + 22%, and a decrease in 23 basins between − 10% and − 33%. One noticeable change is a reduction of the frequency of excess rain in Western Africa, with an increased frequency of floods induced by short rain, consistent with the increase of extreme rainfall observed in this region^3,72^. As shown in Fig. 6, these changes are probably related to a decrease of antecedent soil moisture with decreasing trends detected for this variable in several Western African basins. As noted by Kemter et al.^71^, some flood processes are more coherent in space such as excess rain or long rain that might affect a larger region and affect several basins simultaneously (as seen in West Africa), thus impacting disaster response options. Conversely, a change towards short rains can indicate a more local hydrological response at basin scale but also potentially a change in the magnitude of the floods. Over southern Africa, there is a great variability of the flood generating processes even for neighboring catchments, as shown in Fig. 3, reflecting the strong variability of rainfall in these semi-arid areas and the interplays with catchment characteristics. For southern Africa, only some minor changes between excess/short/long rain are detected and there is not a general tendency.

For only eight basins in Tunisia and South Africa, the Chi-square test identifies a significant change in the distribution of flood drivers between the two time periods. However, it should be noted that for the shortest records with only 20 years, the computation of the test is not very robust. Among them, three stations have a DOR equal to 0, while for the remaining five the DOR ranges from 5 to 100% (for one station). The three stations with zero regulation are intermittent streams, with a frequency of zero flow up to 51% of the record, and they are also characterized by a notable proportion of agricultural areas (i.e., 27%, 84% and 86% of croplands). Even if these basins do not contain a large dam, it is likely that flood processes are influenced by small-scale irrigation facilities^73^. Interestingly, only three out of five basins with DOR > 0 contain a dam in the GrandDam database, highlighting the uncertainties in basin regulation data. Several studies reported important changes in land use in different African regions, mainly an increase in urban and agricultural areas that may influence flood generation processes but also the vulnerability to floods^40,62,67,74–79^. We do not observe such drastic changes in the mechanisms at the origin of floods on a continental scale since these changes in land use probably affect the magnitude of floods at local or plot scales^80^. Furthermore, our analysis focuses on fluvial floods, while several studies suggest that the largest impact of land use change may be observed on urban flood risk, ever increasing in African cities^81^.

## Summary and conclusions

This work provided a continental scale overview of the flood-generating mechanisms across a large sample of basins covering most regions of Africa. A classification scheme was applied to 13,815 flood events that occurred in a wide variety of catchments in terms of size, topography, aridity and land cover. The results indicated that over 75% of floods are driven by excess rainfall and long rainfall episodes. Both processes are related to soil saturation, either before or during a flood event, indicating their role in triggering flood events. This finding has practical consequences related to flood forecasting or the analysis of the impact of climate change on floods. It is indeed necessary to distinguish the influence of soil saturation conditions in addition to that of episodes of intense rainfall. The spatial patterns we detected suggest that climate is the main explanatory factor, with flood-generating processes strongly influenced by aridity, but also modulated by catchment properties. Two main patterns were identified: Western Africa with a dominance of excess rainfall, and North/South Africa but also other semi-arid regions with a mixture of dominant processes. In these regions, one needs to be careful with flood frequency analysis due to the potential presence of a mixture of distributions^82,83^. Overall, no significant changes over time in the dominant flood drivers across regions were detected, except a slight reduction of excess rain in West Africa linked to a decrease of antecedent soil moisture prior to floods. The results confirm to a large extent the findings obtained in other continents, indicating that soil moisture excess is the prevailing driver of flooding^9,48,49^. Yet, the notable difference highlighted that in Africa, compared to other regions, long rains are almost equivalent to the role of excess rainfall to explain the occurrence of floods, in particular for semi-arid regions that are predominant in this continent. This demonstrates the importance of considering the dynamics of soil saturation at different temporal resolutions, in addition to rainfall, to better understand the flood occurrence in different parts of Africa.

## Supplementary Information

Supplementary Figures.

## Data Availability

The flood event database derived from daily river discharge data is available upon request to the corresponding author. Catchment attributes were obtained from the ADHI data set: https://doi.org/10.23708/LXGXQ9. ERA5 rainfall is available from: https://cds.climate.copernicus.eu/cdsapp#!/dataset/reanalysis-era5-pressure-levels?tab=overview. ERA5-land soil moisture from: https://cds.climate.copernicus.eu/cdsapp#!/dataset/reanalysis-era5-land?tab=overview.
