# Supplementary material for: Classification of flood-generating processes in Africa
Source: Sci Rep. 2022 Nov 7;12:18920. doi: 10.1038/s41598-022-23725-5 (PMC9640565; doi:10.1038/s41598-022-23725-5)
Supplement: Supplementary file 1 — Supplementary Figures. [file 41598_2022_23725_MOESM1_ESM.pdf]

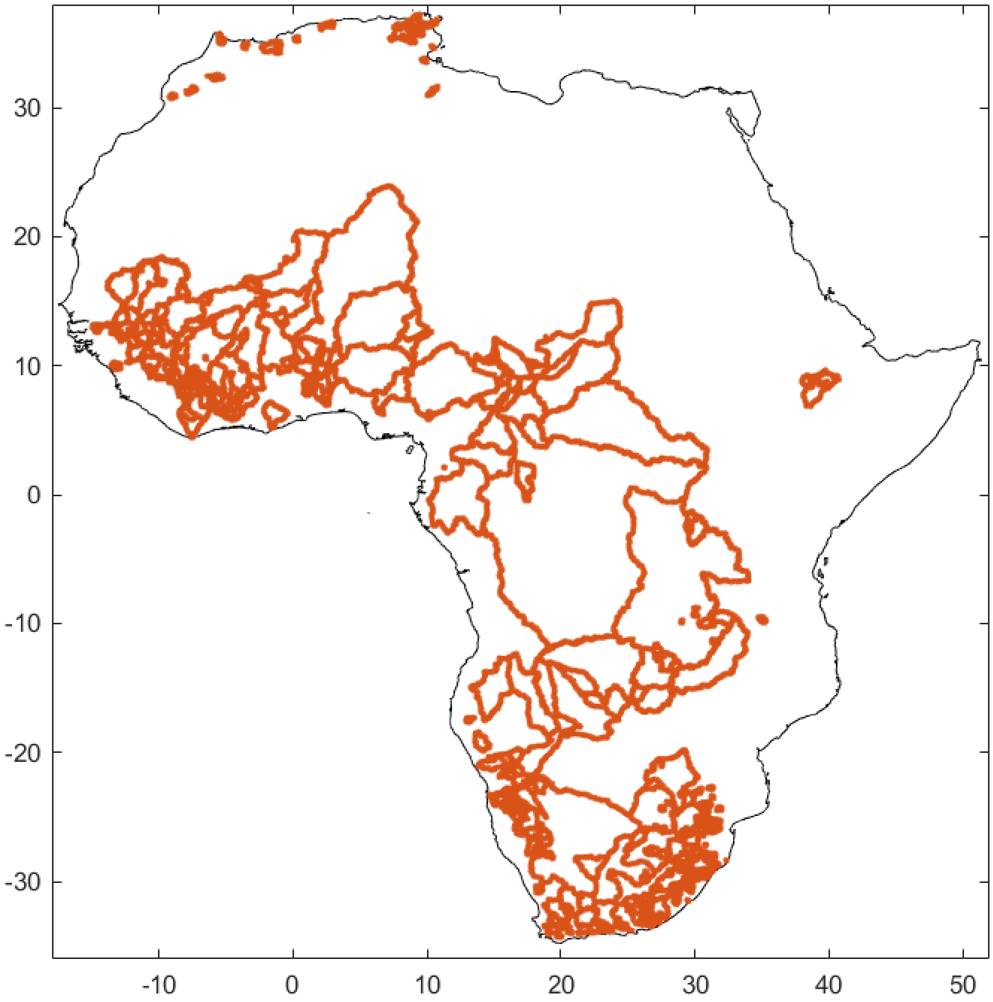

Figure S1: Catchment areas corresponding to the selected stations. The catchment delimitation has been performed with the digital elevation model from the Hydrosheds database (https://www.hydrosheds.org/).

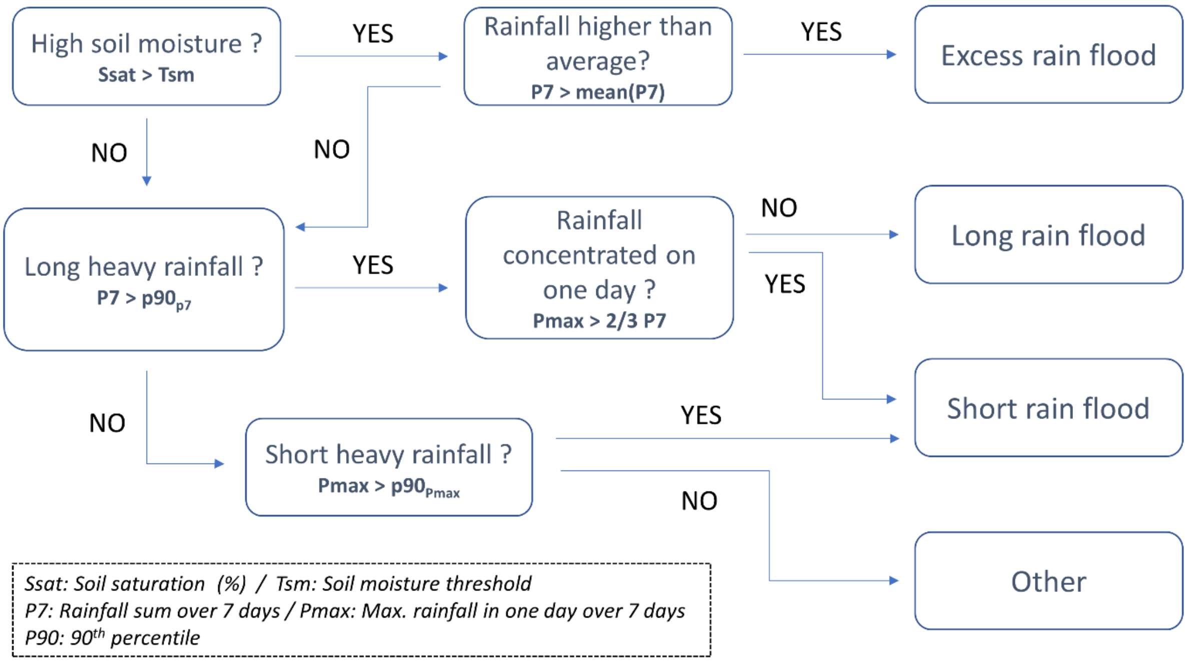

Figure S2: Decision tree for flood classification, modified from Stein et al. 2020
